# Status of HIV Epidemic Control Among Adolescent Girls and Young Women Aged 15–24 Years — Seven African Countries, 2015–2017

**DOI:** 10.15585/mmwr.mm6701a6

**Published:** 2018-01-12

**Authors:** Kristin Brown, Daniel B. Williams, Steve Kinchen, Suzue Saito, Elizabeth Radin, Hetal Patel, Andrea Low, Stephen Delgado, Owen Mugurungi, Godfrey Musuka, Beth A. Tippett Barr, E. Amaka Nwankwo-Igomu, Leala Ruangtragool, Avi J. Hakim, Thokozani Kalua, Rose Nyirenda, Gertrude Chipungu, Andrew Auld, Evelyn Kim, Danielle Payne, Nellie Wadonda-Kabondo, Christine West, Elizabeth Brennan, Beth Deutsch, Anteneh Worku, Sasi Jonnalagadda, Lloyd B. Mulenga, Kumbutso Dzekedzeke, Danielle T. Barradas, Haotian Cai, Sundeep Gupta, Stanley Kamocha, Margaret A. Riggs, Karampreet Sachathep, Wilford Kirungi, Joshua Musinguzi, Alex Opio, Sam Biraro, Elizabeth Bancroft, Jennifer Galbraith, Herbert Kiyingi, Mansoor Farahani, Wolfgang Hladik, Edith Nyangoma, Choice Ginindza, Zandile Masangane, Fortune Mhlanga, Zandile Mnisi, Pasipamire Munyaradzi, Amos Zwane, Sean Burke, Felix B. Kayigamba, Harriet Nuwagaba-Biribonwoha, Ruben Sahabo, Trong T. Ao, Chiara Draghi, Caroline Ryan, Neena M. Philip, Fausta Mosha, Aroldia Mulokozi, Phausta Ntigiti, Angela A. Ramadhani, Geoffrey R. Somi, Cecilia Makafu, Veronicah Mugisha, Julius Zelothe, Kayla Lavilla, David W. Lowrance, Rennatus Mdodo, Elizabeth Gummerson, Paul Stupp, Kyaw Thin, Koen Frederix, Stefania Davia, Amee M Schwitters, Stephen D. McCracken, Yen T. Duong, David Hoos, Bharat Parekh, Jessica E. Justman, Andrew C. Voetsch

**Affiliations:** ^1^Division of Global HIV and TB, Center for Global Health, CDC; ^2^ICAP-Columbia University, New York, New York; ^3^AIDS & TB Programme, Ministry of Health & Child Welfare, Harare, Zimbabwe; ^4^Division of Global HIV and TB, Center for Global Health, CDC Zimbabwe; ^5^PHI/CDC Global Health Fellowship Program; ^6^Ministry of Health Malawi, Lilongwe, Malawi; ^7^Division of Global HIV and TB, Center for Global Health, CDC Malawi; ^8^USAID, Lilongwe, Malawi; ^9^Ministry of Health, Zambia; ^10^Division of Global HIV and TB, Center for Global Health, CDC Zambia; ^11^Ministry of Health, Republic of Uganda; ^12^Division of Global HIV and TB, Center for Global Health, CDC Uganda; ^13^Central Statistical Office, Mbabane, Swaziland; ^14^Ministry of Health, Mbabane, Swaziland; ^15^U.S. Centers for Disease Control and Prevention, Mbabane, Swaziland; ^16^National Health Laboratory Quality Assurance and Training Center, Dar es Salaam, Tanzania; ^17^Tanzania Commission for AIDS, Dar es Salaam, Tanzania; ^18^National Bureau of Statistics, Dar es Salaam, Tanzania; ^19^National AIDS Control Program, Tanzanian Ministry of Health; ^20^Division of Global HIV and TB, Center for Global Health, CDC Tanzania; ^21^Research Coordination Unit, Ministry of Health, Lesotho; ^22^Division of Global HIV and TB, Center for Global Health, CDC Lesotho.

In 2016, an estimated 1.5 million females aged 15–24 years were living with human immunodeficiency virus (HIV) infection in Eastern and Southern Africa, where the prevalence of HIV infection among adolescent girls and young women (3.4%) is more than double that for males in the same age range (1.6%) (*1*). Progress was assessed toward the Joint United Nations Programme on HIV/AIDS (UNAIDS) 2020 targets for adolescent girls and young women in sub-Saharan Africa (90% of those with HIV infection aware of their status, 90% of HIV-infected persons aware of their status on antiretroviral treatment [ART], and 90% of those on treatment virally suppressed [HIV viral load <1,000 HIV RNA copies/mL]) (*2*) using data from recent Population-based HIV Impact Assessment (PHIA) surveys in seven countries. The national prevalence of HIV infection in adolescent girls and young women aged 15–24 years, the percentage who were aware of their status, and among those persons who were aware, the percentage who had achieved viral suppression were calculated. The target for viral suppression among all persons with HIV infection is 73% (the product of 90% x 90% x 90%). Among all seven countries, the prevalence of HIV infection among adolescent girls and young women was 3.6%; among those in this group, 46.3% reported being aware of their HIV-positive status, and 45.0% were virally suppressed. Sustained efforts by national HIV and public health programs to diagnose HIV infection in adolescent girls and young women as early as possible to ensure rapid initiation of ART should help achieve epidemic control among adolescent girls and young women.

The PHIA surveys are nationally representative, household-based surveys funded by the U.S. President’s Emergency Plan for AIDS Relief (PEPFAR) and conducted under the leadership of the respective countries’ ministries of health, CDC, and ICAP at Columbia University (http://www.icap.columbia.edu/). The objectives of the PHIA surveys are to provide national estimates of HIV incidence and subnational estimates of HIV prevalence and viral load suppression to assess the HIV epidemic and the impact of HIV prevention and ART programs in each country. During 2015–2017, PHIA surveys were conducted in Lesotho, Malawi, Swaziland, Uganda, Tanzania Zambia, and Zimbabwe. Each survey used a two-stage cluster sampling design to obtain representative samples of persons living in households within the country. Household members and persons who slept in the household the night before the survey were eligible to participate in the surveys. Persons aged 15–59 years were eligible in all households, and children aged 0–14 years were eligible in one of every two or three households, depending upon the number of participants required to estimate pediatric HIV prevalence. All surveys used comparable questionnaires that included a set of core questions as well as common specimen collection and HIV testing methods.

Data on demographic characteristics, risk behaviors, testing, and treatment history were collected through structured household and individual questionnaires. The surveys included home-based HIV counseling and testing conducted in private locations within or around the home, using each country’s national HIV rapid testing algorithm, and employing CD4 testing technology, with results immediately returned to participants. Awareness of HIV status and current ART use (an indicator of ART coverage at the population level) were determined based on responses provided in the survey questionnaire. HIV viral load testing was conducted using plasma specimens or dried blood spots. Survey data were weighted based on sampling design, nonresponse, and the age and sex distribution of each country’s population. Because each country’s survey weights account for population size, these weights were applied to the pooled data to produce combined estimates for the total population of females aged 15–24 years in the seven countries.

Among the seven countries, 32,273 adolescent girls and young women were eligible for participation; 29,949 (93%) participated in the interview, and 28,152 (94%) of those interviewed participated in the biomarker portion of the survey. The combined prevalence of HIV infection among adolescent girls and young women was 3.6%, ranging from 2.1% in Tanzania to 13.9% in Swaziland (Table). Among HIV-positive adolescent girls and young women, 46.3% reported being aware of their HIV-positive status (range = 40.1% [Zambia] to 70.2% [Swaziland]). Among those who were aware of their HIV-positive status, 85.5% reported current ART use (range = 77.9% [Zambia] to 89.7% [Lesotho]). Among those who reported current ART use, 81.8% were virally suppressed (range = 75.8% [Uganda] to 90.6% [Tanzania]). The overall prevalence of viral load suppression among all adolescent girls and young women with HIV infection, regardless of awareness of HIV-positive status or reported current use of ART, was 45.0%, and ranged from 33.6% in Zambia to 55.5% in Swaziland (Table).

**TABLE:** HIV prevalence, awareness of HIV status, self-reported ART, and viral load suppression among female participants aged 15–24 years in Population-based HIV Impact Assessment (PHIA) surveys — seven Eastern and Southern African countries, 2015–2017

Country	Years survey conducted	HIV prevalence, % (95% CI)	Aware of HIV-positive status, % (95% CI)	Self-reported ART,* % (95% CI)	Viral load suppression among those self-reported on ART,^†^ % (95% CI)	Viral load suppression among all HIV-positive,^§^ % (95% CI)
Zimbabwe	2015–2016	5.9 (5.0–6.7)	48.2 (41.5–55.0)	86.2 (79.4–93.0)	89.0 (83.1–94.9)	47.9 (41.0–54.7)
Malawi	2015–2016	3.4 (2.7–4.2)	55.3 (46.9–63.7)	84.8 (75.9–93.8)	79.6 (67.6–91.6)	49.7 (40.2–59.1)
Zambia	2016	5.7 (4.9–6.5)	40.1 (33.6–46.5)	77.9 (69.3–86.4)	78.1 (67.5–88.7)	33.6 (27.2–39.9)
Uganda	2016–2017	3.3 (2.8–3.82)	44.0 (35.7–52.4)	88.6 (80.9–96.2)	75.8 (64.7–86.9)	44.9 (36.5–53.3)
Swaziland	2016–2017	13.9 (12.1–15.8)	70.2 (64.4–76.1)	79.9 (73.8–85.9)	79.9 (72.7–87.2)	55.5 (49.5–61.5)
Tanzania	2016–2017	2.1 (1.7–2.6)	46.3 (42.8–49.8)	88.2 (77.5–99.0)	90.6 (79.1–100.0)	47.1 (37.3–56.9)
Lesotho	2016–2017	11.1 (9.7–12.5)	61.4 (55.2–67.7)	89.7 (84.8–94.7)	76.4 (69.1–83.7)	50.9 (44.8–57.1)
**Total**	**2015–2017**	**3.6 (3.3–3.9)**	**46.3 (42.8–49.8)**	**85.5 (82.2–88.8)**	**81.8 (77.7–85.9)**	**45.0 (41.6–48.5)**

**Abbreviations:** ART = antiretroviral treatment; CI = confidence interval; HIV = human immunodeficiency virus.

* Percentage who reported antiretroviral treatment among participants who reported being HIV-positive.

^†^ Percentage with viral load suppression (<1,000 HIV RNA copies/mL) among participants who self-reported being HIV-positive and being on antiretroviral treatment.

^§^ Percentage with viral load suppression (<1,000 HIV RNA copies/mL) among participants with HIV-positive test result conducted as part of the PHIA survey, regardless of awareness of diagnosis or reported current use of ART.

## Discussion

The PHIA surveys provide the first population level estimates of viral load suppression for adolescent girls and young women in the seven countries surveyed. Although it is encouraging that among adolescent girls and young women who were aware that they were HIV-positive, 86% reported that they were receiving ART and 82% of those had achieved viral suppression, more remains to be done. Less than half (46.3%) of HIV-positive adolescent girls and young women were aware of their HIV-positive status, which is just over halfway to the 90% UNAIDS target, and based on reported current use of ART, coverage at the population level among adolescent girls and young women with diagnosed HIV infection ranged from 78% to 90%. In Lesotho, Uganda, and Tanzania, self-reported ART use among adolescent girls and young women aware of their HIV-positive status is approaching the 90% target. Although the rate of viral load suppression (45.0%) among all HIV-positive adolescent girls and young women was well below the UNAIDS 73% target, the high rate of viral load suppression among HIV-positive adolescent girls and young women who reported current ART use (82%) is particularly encouraging, suggesting that once these persons receive a diagnosis, national ART programs are successful in initiating and maintaining them on effective ART.

The population of young persons aged 15–24 years in Africa is the fastest-growing youth demographic group globally (*3*). By 2055, the current population of 226 million adolescents and young persons is expected to double (*3*). A rapid and substantial reduction in HIV incidence in this population is critical to achieve epidemic control by 2030.

PEPFAR’s DREAMS (Determined, Resilient, Empowered, AIDS-free, Mentored, and Safe) initiative is a public-private partnership aimed at reducing the impact of HIV on adolescent girls and young women by engaging them, their families, and their communities through programs aimed at addressing the economic, cultural, legal, and behavioral drivers of new HIV infections in this population (*4*). DREAMS interventions consist of programs aimed at risk reduction for HIV-negative adolescent girls and young women (*4*,*5*). Because a significant percentage of HIV-positive adolescent girls and young women do not know their status, strategies for identifying effective and innovative case finding linked to same day treatment in this population are needed and would complement the existing DREAMS strategies (*6*).

The findings in this report are subject to at least one limitation. HIV status-awareness and ART coverage are based on participants’ responses to the survey questionnaire. These two indicators might be underestimated if HIV-positive participants were unwilling to report knowing their HIV status, which might be the case among adolescents in particular (*7*). Multiplying the three 90/90/90 target measures from this analysis together (46.3% aware of HIV-positive status x 85.5% self-reported ART use x 81.8% viral suppression among those on ART) produces a viral load suppression prevalence among HIV-positive adolescent girls and young women on ART of 32.4%. This is lower than the 45.0% observed via biomarker viral load suppression among all HIV-positive adolescent girls and young women, suggesting underreporting in the measurement of the first two targets. Absent underreporting, virtually all of the 46.3% of HIV-positive adolescent girls and young women reporting awareness of their HIV-positive status would need to be on ART and suppressed to achieve the 45.0% overall viral load suppression. This is unlikely given that 14.5% of those who were aware of their status did not report current ART use, and a more likely explanation is that there is some level of underreporting of both knowledge of status and ART use. All HIV-positive blood specimens collected for the PHIA surveys will be tested for the presence of selected antiretroviral medications, based on the national treatment guidelines, to provide additional measures of ART coverage. Although the results of the ART testing are pending, overall viral load suppression is based on objective measures and is, therefore, not subject to the same sources of underestimation.

There has been notable progress toward overall HIV epidemic control in countries in this region, as documented by PHIA survey results (2015–2016) from Malawi, Zambia, and Zimbabwe, which found that 62.0% of all HIV-positive adults aged 15–59 years were virally suppressed (*8*). In Swaziland, the prevalence of viral load suppression among HIV-positive adults aged 18–49 years more than doubled from 34.8% in 2011 to 71.3% in 2017, and a 44% decline in HIV incidence was observed over the same period (*9*). In contrast to these successes in the general adult population, the 45% prevalence for viral load suppression among adolescent girls and young women is well below the 73% target, suggesting the strategies that have been more broadly successful in initiating and keeping adults with HIV on ART are less successful in this population. Even as significant progress has been made toward achieving the 90/90/90 targets in these countries, additional, targeted strategies are needed to reach some groups, particularly adolescent girls and young women.

**Summary**

**What is already known about this topic?**

In 2016, an estimated 1.5 million adolescent girls and young women were living with HIV infection in Eastern and Southern Africa, where HIV prevalence among adolescent girls and young women is more than twice that of their male peers.

**What is added by this report?**

Analysis of data from Population-based HIV Impact Assessment surveys conducted during 2015–2017 in seven countries in Eastern and Southern Africa found that the prevalence of HIV infection among adolescent girls and young women was 3.6%. Among those who were HIV-positive, 46.3% reported being aware of their status, and among those aware of their HIV-positive status, 85.5% reported current antiretroviral treatment (ART) use. Overall, viral load suppression among HIV-infected adolescent girls and young women, regardless of status awareness or current use of ART, was 45.0%, well below the UNAIDS target of 73%.

**What are the implications for public health practice?**

There is a need to design, implement, and evaluate strategies aimed at ensuring HIV-positive adolescent girls and young women know their HIV status and are on ART treatment to improve their immunity status and reduce transmission to others.
